# Distinct profiles of reactive and proactive aggression in adolescents: associations with cognitive and affective empathy

**DOI:** 10.1186/s13034-016-0141-4

**Published:** 2017-01-06

**Authors:** Felix Euler, Célia Steinlin, Christina Stadler

**Affiliations:** Department of Child and Adolescent Psychiatry, Psychiatric University Clinics Basel, Schanzenstrasse 13, 4056 Basel, Switzerland

**Keywords:** Empathy, Aggression, Basic Empathy Scale, Juvenile detention, Adolescents

## Abstract

**Background:**

Aggression comprises a heterogeneous set of behavioral patterns that aim to harm and hurt others. Empathy represents a potential mechanism that inhibits aggressive conduct and enhances prosocial behavior. Nevertheless, research results on the relationship between empathy and aggression are mixed. Subtypes of aggressive behavior, such as reactive and proactive aggression might be differently related to empathy. The aim of the present study was to investigate the interrelations of cognitive and affective empathy with reactive and proactive aggression.

**Methods:**

We recruited a sample of 177 (33% female, M age 15.6) adolescents from socio-educational and juvenile justice institutions and a community sample of 77 (36% female, M age 13.1) adolescents from secondary schools. Using bivariate correlation analysis and hierarchical multiple regression analysis, we firstly investigated associations between cognitive and affective empathy and reactive and proactive aggression. Subsequently, we performed cluster analysis to identify clusters of adolescents with meaningful profiles of aggressive behavior and compared derived clusters on measures of empathy. We applied the Basic Empathy Scale and the Reactive-Proactive Aggression Questionnaire.

**Results:**

Bivariate analysis and hierarchical regression analysis showed that cognitive and affective empathy were negatively associated with proactive aggression, but not with reactive aggression. Cluster-analysis revealed three clusters of adolescents with distinct aggression profiles: a cluster with elevated scores on reactive and proactive aggression, a clusters with high scores on reactive aggression only, and a low aggression cluster. Cluster comparisons revealed that the reactive-proactive aggression cluster showed significantly lower scores on cognitive and affective empathy than both other clusters. Results further indicated that within the reactive-proactive aggression cluster, girls did not differ significantly from boys in empathy.

**Conclusions:**

The present study extends previously published findings, and possibly explains conflicting results in prior research. Our results indicated that cognitive and affective empathy are reduced in adolescents with high levels of reactive and proactive aggression. Our study may contribute to the development of tailored clinical interventions for different aggression clusters.

## Background

Aggression is usually defined as behavior deliberately aimed to harm individuals and/or objects [1]. One construct of interest related to the development and manifestation of pathologic aggression is empathy. Empathic individuals are thought to use information about emotional states in others to constrain potentially harmful behaviors and to inhibit antisocial and aggressive acts [2, 3]. The experience of empathy is associated with helping and comforting others [4]. Adequate empathic responding is an important aspect of reciprocal human relationships and represents an essential component of moral and social development [5]. Empathy is defined as a complex interpersonal phenomenon in which observation, memory, knowledge, and reasoning are combined to give insights into the thoughts and feelings of others [6]. It comprises the perception and the affective response of the emotional state of someone else [7, 8]. Contemporary conceptualizations of empathy have emphasized the distinction of cognitive and affective components [5, 9]. According to Jolliffe and Farrington [10] affective empathy is specified as ‘affect congruence’ and cognitive empathy as ‘the understanding of another’s emotions’. The distinction of cognitive and affective empathy components represents a promising step to disentangle the multilevel construct of human empathy.

Despite the assumptions about the relevance of empathy deficits for the development and manifestation of aggressive behavior, meta-analyses indicate that empirical research does not clearly support a significant relationship between empathy and aggression. In their meta-analysis, Vachon et al. [11] concluded that empathy and aggression share only a small amount of variance. Earlier, Lovett and Sheffield [12] summarized that findings on the association between affective empathy and aggression in children and adolescents are inconsistent. Eisenberg et al. [13] reported that empathy is only moderately associated with aggressive behavior. Interestingly, recent research revealed that cognitive and affective empathy subcomponents are differently associated with subtypes of disruptive behavior in children and adolescents [14–16]. Therefore, the expected association between empathy and aggression may only apply to specific forms of aggression.

One important differentiation of aggressive behavior is the distinction between reactive and proactive aggression introduced by Dodge and Coie [17]. Reactive aggression is described as an impulsive response to a perceived threat or provocation, often associated with high emotional arousal, anxiety, and anger. Proactive aggression is described as instrumental, organized, cold-blooded, and motivated by the anticipation of reward [17, 18]. A number of studies have documented different associations of the two aggression subtypes with cognitive and affective variables [19]. Based on the motivational underpinnings of the two subtypes of aggression, it can be assumed that empathy is differentially involved in the inhibition of reactive and proactive aggression. Accordingly, it has been proposed that for reactive aggression, emotional over-arousal disturbs inhibition mechanisms usually triggered by empathy [12]. Neurodevelopmental models of empathy [20–22] further substantiate this assumption. These models emphasize that adequate emotion regulation is a prerequisite for the experience of empathy. Because deficient emotion regulation is a core feature of reactive aggression, empathy is less likely to be involved in the inhibition of this subtype of aggression. In contrast, planned and controlled acts of aggression are more likely to be inhibited by earlier experiences of empathy. In line with these assumptions, Kimonis et al. [23] showed that reduced responding to emotional stimulation is associated with proactive aggression in non-referred girls and boys. Moreover, proactive aggression in the form of bullying has been associated with lower levels of affective empathy in male and female adolescents [24]. Nonetheless, Feshbach and Feshbach [25] have argued that empathy hinders both types of aggression. Moreover, a recent investigation with healthy adults showed that both types of aggression are negatively associated with cognitive and affective empathy [26]. A study with children with autism spectrum disorder and healthy controls indicated that empathy is associated with reactive but not with proactive aggression [27]. Overall, empirical findings on the association between empathy and reactive and proactive aggression are heterogeneous. At present it remains unclear if empathy is equally associated with neither, one, or both forms of aggression in children and adolescents.

One major issue in research investigating associations of reactive and proactive aggression is the high correlation between the two aggression subtypes. Across different samples investigations have reported correlations between .4 and .9 [28]. Moreover, individuals showing proactive aggression only, are usually difficult to identify. While primarily reactive aggressive individuals have often been characterized, individuals high on proactive aggression are usually also high on reactive aggression [29]. Consequently, the value of the differentiation between reactive and proactive aggression has been questioned and it has been argued that proactive aggression is simply an indication of a more severe aggressive behavioral pattern [30]. Therefore, identifying correlates of reactive and proactive aggression with other variables might not be sufficient to support the usefulness of the dichotomy in clinical practice. It has been suggested that it is important to apply methods controlling for the co-occurrence of each aggression subtype. Recent research has applied person-centered group comparisons to solve this issue. These studies have compared individuals with meaningful profiles of reactive and proactive aggression [29, 31, 32].

Although the reactive-proactive aggression distinction has been acknowledged in some studies investigating the empathy-aggression relationship, studies comparing cognitive and affective facets of empathy between clusters of adolescents with meaningful aggression profiles are still scarce. Mayberry and Espelage [32] applied this approach, but did not find the expected differences in empathy between identified aggression clusters. One limitation of the study by Mayberry and Espelage [32], and most other studies investigating the empathy-aggression relationship, has been that participants with elevated levels of aggression were not included in the samples. This has made it difficult to draw conclusions about the involvement of empathy dysfunction in pathologic aggressive individuals [12]. Especially for the development of clinical interventions that aim to reduce aggressive behavior, it seems important to understand if empathy is related to subtypes of aggressive behavior and which empathy subcomponents should be the focus of such intervention programs.

Another important topic regarding the empathy-aggression relationship is gender. Girls usually show less severe aggressive behavior [33], and are less likely to develop aggression related disorders [34]. For reactive and proactive aggression, recent studies also report significant gender differences with boys scoring higher than girls on both types of aggression [35, 36]. Further, research indicated that associations of reactive and proactive aggression with future psychopathology differed between boys and girls [37]. Gender differences have also consistently been reported for empathy [2]. Girls usually score higher on self and other-reported measures of cognitive and affective empathy [10, 38]. In adolescent samples, gender differences are usually more distinct for affective than for cognitive empathy [39, 40]. Of notice, studies that have investigated gender differences in empathy mostly did not acknowledge levels of aggressive behavior within their subjects. An interesting question is, whether girls and boys with comparable levels and similar profiles of aggressive behavior differ in empathy in a way non-aggressive youth do. To our knowledge, differences between girls and boys within clusters of adolescents with meaningful aggression profiles have not been investigated yet.

### Aim of the present study

Since successful social interactions during adolescence have a large impact on socio-emotional functioning, a better understanding of the interrelation between empathy and aggression during that age period appears especially relevant and is an important subject of investigation. Given the heterogeneous findings and limitations of previous investigations on the aggression-empathy relationship, the present study aimed to further advance the knowledge in the field by investigating the following research questions: (1) Are cognitive and affective empathy associated with reactive and proactive forms of aggression? (2) Do clusters of aggressive adolescents, with meaningful aggression profiles differ in cognitive and affective empathy? (3) Do girls and boys within aggression clusters differ in cognitive and affective empathy? Based on previous empirical findings and theoretical assumptions regarding the motivational underpinnings of reactive and proactive aggression, for our first study question we hypothesized that cognitive and affective empathy are negatively associated with proactive aggression but not with reactive aggression. For our second study question, we firstly derived clusters of adolescents with distinct aggression profiles. We expected to find a low aggression, a reactive aggression only, and a reactive-proactive aggression cluster. We hypothesized to find significant differences between emerging aggression clusters on cognitive and affective empathy. For our third study question we compared girls and boys within derived aggression clusters on cognitive and affective empathy. In line with previous research showing gender differences in empathy we hypothesized that within the low aggression and the reactive aggression only cluster, girls differ significantly from boys on affective empathy. Contrary, we expected that in adolescents with elevated levels of proactive aggression affective empathy to be reduced, irrespective of gender. Therefore, we assumed to find smaller and non-significant differences in affective empathy between girls and boys within the cluster of adolescents with elevated levels of proactive aggression. Since previous research did not consistently report gender differences on cognitive empathy in adolescents, we hypothesized that girls and boys within all aggression clusters show similar scores on cognitive empathy.

## Methods

### Participants

A total sample of 254 adolescents (35% female, M age 14.9) between the age of 12 and 18 years participated in the survey. Of the total sample 177 (33% female, M age 15.6) were recruited from socio-educational and juvenile justice institutions in the German speaking part of Switzerland. We recruited adolescents from these institutions because we expected to find elevated levels of reactive and proactive aggression in this sample. Adjudicated youth generally show higher levels of aggressive behavior than age-equivalent adolescents in the general population [41, 42]. Additionally a community sample of 77 adolescents (36% female, M age 13.1), living at home with their parents, were recruited from Swiss secondary schools. A sample size estimation was performed a priori for our study questions that were tested using regression models. The estimated minimum sample size required was N = 127. Since we were planning to conduct a follow up study with our sample, and expected a drop-our rate of 50%, we collected a total of 254 data sets. Participating socio-educational and juvenile justice institutions were all accredited by the Swiss Ministry of Justice. Adolescents were admitted to these institutions by way of either criminal (46.6%) or civil (54.4%) law. Hospitalization by civil law occurred if adolescents were no longer able to live in their family or environment of origin due to severe psychological or behavioral problems or precarious life conditions. At the time of testing most of the institutionalized participants were attending regular secondary school (59.4%) or participated in vocational training (5.5%). About one-third visited school inside the facilities (27.6%). Some were not involved in any gainful activity at the time of testing (7.5%). Adolescents with insufficient German language skills were excluded a priori from the study. Missing data were replaced using the Expectation–Maximization function in SPSS. Five data sets from adolescents recruited in socio-educational and juvenile justice institutions and three data sets from the community sample had to be excluded from the analysis because of a large number of missing items on the questionnaires. Further, four subjects recruited in socio-educational and juvenile justice institutions and one subject from the community sample were excluded after having reported that they had marked items randomly or because they refused to follow instructions during data assessment. A total of N = 241 (N = 168 institutionalized adolescents; N = 73 community sample) data sets were used in the statistical analysis.

### Procedure

In a first step, we contacted child welfare and juvenile justice institutions and secondary schools in the German speaking part of Switzerland. If an institution agreed to participate, adolescents, caseworkers, and/or parents were informed about the project. If written informed consent for the survey was given by the adolescents and the person entitled to their custody, the research team visited the institution and participating adolescents filled in questionnaires during group sessions. Investigators were always present during test sessions to answer questions. Information disclosed by the youths remained confidential and feedback was given only if the adolescent consented. Subjects received a movie theater gift voucher for participation in the study. Ethical approval for the study was obtained by the Institutional Review Board of the University of Basel, Switzerland.

### Instruments

#### Empathy

Adolescents completed the Basic Empathy Scale [BES; 10]. The BES is a self-report instrument that comprises the subscales ‘cognitive empathy’ (9 items) and ‘affective empathy’ (11 items) and a ‘total empathy’ (20 items) scale. Previous investigations supported convergent, discriminant, and predictive validity of the BES across age and gender [10, 39, 40]. We administered a German version of the BES. The original BES was translated and back-translated by native English and German speakers. Discrepancies were discussed and corrected. Adolescents rated how much each item applied to them on a 5-point Likert scale (‘strongly disagree’, ‘disagree’, ‘neither agree nor disagree’, ‘agree’, ‘strongly agree’). For the current sample, the BES affective (α = .77), the cognitive (α = .75), and the total empathy scale (α = .82) showed sufficient internal consistencies.

#### Aggressive behavior

The Reactive-Proactive Aggression Questionnaire [RPQ; 18] was applied to assess subtypes of aggression. The RPQ is a self-report questionnaire that uses a three-point Likert scale (‘never’, ‘sometimes’, ‘often’) and comprises the subscales ‘reactive aggression’ (12 items) and ‘proactive aggression’ (11 items), and a ‘total aggression’ (23 items) scale. The RPQ assesses both types of aggression reliably and validly and factor analyses have confirmed the two-factor conceptualization of the items [43]. In the present study, adolescents completed a German version of the RPQ. The original version of the RPQ was translated and back-translated by native English and German speakers. Discrepancies were discussed and corrected. Internal consistencies for the reactive aggression (α = .85), the proactive aggression (α = .87), and the total RPQ scale (α = .91) of the German RPQ version in the present study were excellent.

### Statistical analyses

To address our first research question we ran bivariate correlation and hierarchical multiple regression analysis. We primarily calculated bivariate correlations between the main study variables, cognitive, affective, and total empathy, reactive, proactive, and total aggression. Age and gender were also included in the bivariate analysis. Subsequently, we performed hierarchical multiple regression analyses to determine whether cognitive and affective empathy improved prediction of reactive and proactive aggression beyond that afforded by gender, age, and reactive or proactive aggression respectively. For regression models we tested independence of errors using the Durbin-Watson statistics. Homogenity of variance was evaluated using the Variance Inflation Factor (VIF). The VIF measures the impact of collinearity among the variables in a regression model. With the use of a *p* < .001 criterion for Mahalanobis distance, we screened each regression model for outliers. To investigate our second study question, we firstly performed cluster analysis to identify clusters of adolescents with distinct aggression profiles. Subsequently we compared emerging aggression clusters on cognitive and affective empathy. For cluster derivation we performed the TwoStep cluster analysis (CA) procedure offered by SPSS. This procedure is a scalable CA algorithm developed to automatically find the optimal number of clusters in large datasets. In a first step, the procedure calculates the Bayesian information criterion (BIC) for each number of clusters in a given range. In a second step, a model-based hierarchical technique refines the initial number by estimating the ratio of distance between clusters. We used the PRQ reactive and proactive aggression subscales as clustering variables. Because at present no established cutoff scores are available for the RPQ, we interpreted scores of derived clusters in reference to empirical investigations that have used the RPQ in adolescent samples [18, 31, 44]. We ran Chi square tests to analyze distribution of categorical variables across identified clusters. For group comparisons between derived clusters on measures of interest, we used univariate analysis of covariance (ANCOVA) and included age and gender as covariates. Bonferroni corrections were applied for post hoc multiple comparisons between clusters. To address our third study question, if girls and boys within aggression clusters differ in cognitive and affective empathy, we used independent samples t tests. We compared girls and boys within each aggression cluster on reactive, proactive, and total aggression, and on cognitive, affective, and total empathy. If Levene’s test did not confirm homogeneity of variance for between gender comparisons, reported results are adjusted for inequality of variances. Because of the large number of statistical tests, alpha was set to *p* < .01 as indicator of significance. We used the IBM-SPSS software package, Version 22 (IBM SPSS Inc., Chicago, USA) for the statistical analysis. Prior to our analysis, we screened data for violation of assumptions. Explorative analysis suggested that normality was a reasonable assumption for the main study variables. Normality was tested via the Shapiro–Wilk test.

## Results

### Bivariate and hierarchical regression analysis

Table 1 indicates descriptive statistics separately for the institutionalized, the community, and the total study sample on measures of interest. As expected, institutionalized adolescents scored higher than adolescents from the community sample on reactive, proactive, and total aggression, and lower on cognitive, affective, and total empathy. Results of the bivariate analysis for the main study variables are depicted in Table 2. The zero-order Pearson *r* indicated that proactive and total aggression correlated negatively and significantly with cognitive, affective, and total empathy. Associations between reactive aggression cognitive, affective, and total empathy were not significant. Aggression subtypes and empathy subcomponents correlated significantly with each other. Bivariate analysis also revealed that age was significantly correlated with total empathy, reactive aggression, proactive aggression, and total aggression. Gender was significantly associated with affective empathy, total empathy, and with proactive aggression. Next, we conducted two hierarchical multiple regression models. In the first regression model we entered proactive aggression and in the second model reactive aggression as the dependent variable. In each model age, gender, and either reactive or proactive aggression were entered at stage one to control for the influence of these variables. Cognitive empathy and affective empathy were entered at stage two. Evaluation of the assumptions indicated that linearity, independence of errors, and homoscedasticity of residuals were acceptable for each regression model. No outliers were identified for any of the regression models with the use of a *p* < .001 criterion for Mahalanobis distance.

**Table 1:** Descriptive statistics for aggression and empathy subscales

	Institutionalized sample (n = 168)		Community sample (n = 73)		Total sample (n = 241)		Range	
	M	(SD)	M	(SD)	M	(SD)	Min	Max
*Aggression (RPQ)*								
Reactive aggression	11.79	(5.20)	7.60	(3.82)	10.52	(5.19)	0	24
Proactive aggression	5.92	(5.10)	2.04	(2.05)	4.75	(4.75)	0	21
Total aggression	17.71	(9.35)	9.64	(5.07)	15.27	(9.07)	0	44
*Empathy (BES)*								
Cognitive empathy	36.05	(5.01)	37.62	(3.46)	36.53	(4.64)	11	54
Affective empathy	33.95	(6.68)	37.49	(3.39)	35.02	(7.23)	19	45
Total empathy	70.00	(9.66)	75.11	(10.20)	71.55	(10.09)	40	99

*BES* Basic Empathy Scale, *RPQ* Reactive-Proactive Aggression Questionnaire

**Table 2:** Bivariate analysis for the main study variables (n = 241)

		1	2	3	4	5	6	7
1.	BES affective empathy	−						
2.	BES cognitive empathy	.42**	–					
3.	BES total empathy	.91**	.76**	–				
4.	RPQ reactive aggression	−.11	−.14	−.14	–			
5.	RPQ proactive aggression	−.31**	−.29**	−.35**	.67**	–		
6.	RPQ total aggression	−.22*	−.23**	−.26**	.92**	.90**	–	
7	Age	−.16	−.13	−.18*	.34**	.35**	.38**	
8	Gender^a^	.28**	.13	.26**	−.07	−.19*	−.14	−.09

Pearson coefficients (2-tailed) are given

*BES* Basic Empathy Scale, *RPQ* Reactive-Proactive Aggression Questionnaire

* p < .01, ** p < .001

^a^Negative coefficients indicate higher scores for boys

#### Proactive aggression

Table 3 depicts the raw and standardized regression coefficients of the predictors, their squared semipartial correlations and their structure coefficients for the regression model with the dependent variable proactive aggression after entry of all five independent variables (IVs). After stage two, with all IVs in the equation, the model was statistically significant, F_(5,235)_ = 51.83, *p* < .001, and accounted for approximately 52% of the variance in proactive aggression (*R* ^2^ = .52, Adjusted *R* ^2^ = .51). After stage one with reactive aggression, gender, and age in the equation, the model was also statistically significant, F_(3,237)_ = 72.07 *p* < .001, and accounted for approximately 48% of the variance in proactive aggression (*R* ^2^ = .48, Adjusted *R* ^2^ = .47). Introducing cognitive and affective empathy explained an additional 5% of variation in proactive aggression, and this change in *R* ^2^ was significant (*p* < .001). Proactive aggression was significantly and uniquely predicted by reactive aggression and affective empathy. Squared semipartial correlations indexed that the unique variance explained by reactive aggression was substantial, the unique variance explained by affective empathy was low.

**Table 3:** Hierarchical multiple regression analysis predicting proactive aggression (n = 241)

Variables	B	β	*sr*_unique_^2^	R	R^2^	ΔR^2^
Step 1				.691	.477	.470
Age	.32	.73	.03			
Gender	−1.32*	1.79	.04			
Reactive aggression	.56**	−2.08	.39			
Step 2				.724	.524	.514
Age	.25	.10	.02			
Gender	−.78	−.08	.01			
Reactive aggression	.54**	.59	.39			
Cognitive empathy	−.11	−.16	.02			
Affective empathy	−.11*	−.11	.04			

*sr* ^*2*^ = squared semipartial correlation

* p < .01, ** p < .001

#### Reactive aggression

Table 4 summarizes the raw and standardized regression coefficients of the predictors, their squared semipartial correlations, and their structure coefficients for the regression model with the dependent variable reactive aggression after inclusion of all six IVs. After stage two, with all independent variables in the equation, the model became statistically significant, F_(5,235)_ = 41.64, *p* < .001, and accounted for approximately 47% of the variance in reactive aggression (*R* ^2^ = .47, Adjusted *R* ^2^ = .46). After stage one with proactive aggression, gender and age in the equation the model also reached statistical significance, F_(3,237)_ = 67.24, *p* < .001, and accounted for approximately 46% of the variance in reactive aggression (*R* ^2^ = .46, Adjusted *R* ^2^ = .45). Introducing cognitive and affective empathy explained an additional 1% of variation in reactive aggression, this change in *R* ^2^ was not significant. Reactive aggression was significantly predicted by proactive aggression only. The unique variance explained by proactive aggression indexed by the squared semipartial correlations was substantial.

**Table 4:** Hierarchical multiple regression analysis predicting reactive aggression (n = 241)

Variables	*B*	*β*	*sr*_*unique*_^*2*^	*R*	R^2^	ΔR^2^
Step 1				.678	.460	.453
Age	.35	.13	.03			
Gender	.66	.06	.01			
Proactive aggression	.69**	.63	.39			
Step 2				.685	.470	.458
Age	.36	.13	.03			
Gender	.39	.04	.00			
Proactive aggression	.72**	.66	.39			
Cognitive empathy	.02	.02	.00			
Affective empathy	.07	.10	.01			

*sr* ^*2*^ = squared semipartial correlation

** p < .001

### Cluster derivation

The two-step cluster procedure indicated a three-cluster solution. With a BIC change of −40.06 between the two- and three-cluster solutions and a ratio of distance measure of 3.19, the algorithm judged the three-cluster solution to be the best fit for our data. The three-cluster solution represented a better fit than the four-cluster solution with a BIC change between the three- and four-cluster solution of 2.47 and a ratio of distance measure of 1.20.

Table 5 shows the mean scores for the aggression and the empathy subscales for the three derived clusters and indicates results of post hoc Bonferroni adjusted group comparisons. According to the aggression profiles, the first cluster designated a ‘reactive-proactive aggression’ cluster, the second cluster a ‘reactive aggression’ cluster, and the third cluster a ‘low aggression’ cluster. These labels are further used to refer to the respective clusters in this manuscript. The reactive-proactive aggression cluster had higher scores on reactive, proactive, and total aggression than both other clusters. The reactive aggression cluster scored higher than the low aggression cluster on reactive, proactive, and total aggression. We subsequently interpreted scores of the derived clusters on reactive and proactive aggression in references to the mean scores of the male community sample investigated by Raine et al. [18], the male sample of detained juveniles assessed by Colins [31], and the sample of juvenile delinquents studied by Cima et al. [44]. The reactive-proactive aggression cluster scored more than 1 SD above the mean scores on the reactive and the proactive aggression scale compared to all of the abovementioned study samples. The reactive aggression cluster scored more than 1 SD above the mean score of the comparison samples investigated by Raine et al. [18] and Colins [31] on the reactive aggression scale, but not on the proactive aggression scale. The low aggression cluster scored within the range of 1 SD on both aggression scales in reference to the abovementioned comparison samples.

**Table 5:** Aggression and empathy scores for identified clusters and results of cluster comparisons

	Re pro AGG (n = 62)		Re AGG (n = 101)		Low AGG (n = 78)		Re pro AGG versus re AGG	Re pro AGG versus low AGG	Re AGG versus low AGG
	M	(SD)	M	(SD)	M	(SD)	*p*	*p*	*p*
*Aggression (RPQ)*									
Reactive aggression	16.06	(4.00)	11.52	(2.60)	4.81	(1.80)	<0.001	<0.001	<0.001
Proactive aggression	11.53	(3.60)	3.21	(2.19)	1.35	(1.39)	<0.001	<0.001	<0.001
Total aggression	27.60	(6.14)	14.74	(3.22)	6.15	(2.50)	<0.001	<0.001	<0.001
*Empathy (BES)*									
Cognitive empathy	34.18	(5.15)	37.30	(4.23)	37.40	(4.11)	<0.001	<0.01	ns
Affective empathy	31.74	(5.73)	36.32	(7.39)	35.95	(7.35)	<0.01	=0.032	ns
Total empathy	65.91	(7.80)	73.61	(10.08)	73.35	(10.11)	<0.001	<0.01	ns

*BES* Basic Empathy Scale, *RPQ* Reactive-Proactive Aggression Questionnaire, *re pro AGG* reactive-proactive aggression cluster, *re AGG* reactive aggression cluster, *low AGG* low aggression cluster

*p* values refer to Bonferroni adjusted post hoc comparisons between identified aggression clusters with age and gender as covariates

### Cluster comparisons

Firstly, we analyzed distribution of gender across derived clusters. Of the 87 girls, 18.4% (N = 16) were in the reactive-proactive aggression cluster, 44.8% (N = 39) in the reactive aggression cluster, and 36.8% (N = 32) in the low aggression cluster. Of the 154 boys, 29.9% (N = 46) were in the reactive-proactive aggression cluster, 40.3% (N = 62) in the reactive aggression cluster and 29.9% (N = 46) in the low aggression cluster. Chi square tests indicated that the gender distribution did not differed significantly between clusters (χ^2^ = 3.95, N = 241, *p* = .139). Next we tested distribution of participants living in institutions and participants from the community sample across clusters. Of the 168 institutionalized adolescents, 36.3% (N = 61) were in the reactive-proactive aggression cluster, 40.5% (N = 68) in the reactive aggression cluster, and 23.2% (N = 39) in the low aggression cluster. Only 1.4% (N = 1) of the community sample were in the reactive-proactive aggression cluster, 45.2% (N = 33) in the reactive aggression cluster, and 53.4% (N = 39) in the low aggression cluster. As expected Chi square tests indicated significant differences in the distribution of adolescents living in institutions and adolescents living at home (χ^2^ = 38.77, N = 241, *p* < .001). Age differed significantly between aggression clusters (*F*_(2,238)_ = 18.98, *p* < .001; η^2^ = .14). The reactive-proactive aggression cluster was significantly older (M age 15.9), than the reactive aggression (M age 14.7), and the low aggression (M age 14.2) cluster. The later two did not differ significantly in age.

**Fig. 1:**
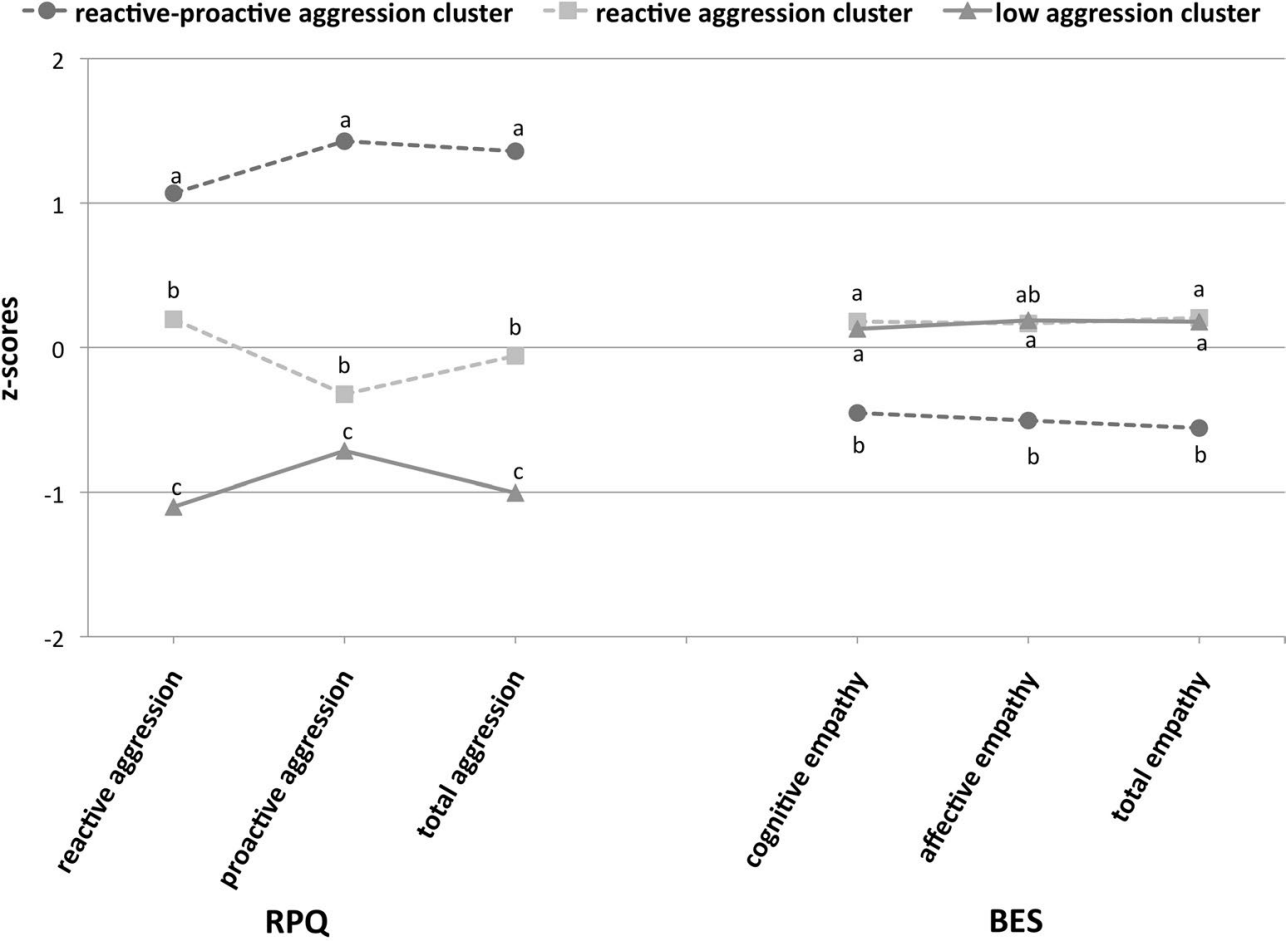
Z-scores for aggression and empathy subscales separately for derived aggression clusters. Subscripts (*a*, *b*, *c*) denote significant differences between clusters in Bonferroni adjusted post hoc comparisons with age and gender as covariates (*p* < 0.01). *BES* Basic Empathy Scale, *RPQ* Reactive-Proactive Aggression Questionnaire

Secondly, we performed univariate ANCOVAs to compare aggression clusters on reactive, proactive, and total aggression, and on cognitive, affective, and total empathy. Because bivariate analysis had indicated significant associations of age and gender with the measures of interest, both were included as covariates for group comparisons. Figure 1 shows standardized z-scores for aggression and empathy separately for each derived aggression cluster. Subscripts in Fig. 1a–c denote significant differences between clusters in Bonferroni adjusted post hoc comparisons with age and gender as covariates. Results of univariate ANCOVAs indicated a significant effect of aggression cluster for reactive (*F*_(2,236)_ = 240.42, *p* < .001; η^2^ = .67), proactive (*F*_(2,236)_ = 276.90, *p* < .001; η^2^ = .70), and total aggression (*F*_(2,236)_ = 408.56, *p* < .001; η^2^ = .78). The covariates age and gender did not become significant for aggression measures. Post-hoc Bonferroni adjusted comparisons revealed significant differences for all between cluster comparisons for reactive, proactive, and total aggression. In line with our expectations, univariate ANCOVAs also revealed a significant effect of aggression cluster for affective (*F*_(2,236)_ = 5.61, *p* < .01; η^2^ = .05), cognitive (*F*_(2,236)_ = 8.70, *p* < .001; η^2^ = .07) and total empathy (*F*_(2,236)_ = 9.69, *p* < .001; η^2^ = .08). For affective and total empathy the covariate gender became significant. Post-hoc Bonferroni adjusted comparisons revealed that the reactive-proactive aggression cluster differed significantly from the reactive aggression cluster, and from the low aggression cluster on cognitive and total empathy. On affective empathy the reactive-proactive aggression cluster differed significantly only from the reactive aggression cluster. The reactive aggression cluster and the low aggression cluster did not differ significantly on cognitive, affective, and total empathy.

### Gender comparison

To answer our last study question, we assessed gender differences in empathy within each derived aggression cluster. Table 6 depicts mean sores for aggression and empathy separately for boys and girls within each aggression cluster and for the total study sample. Independent samples t tests revealed that within the total study sample girls had significant lower scores on proactive aggression (*t*_(239)_ = 3.27, p < 0.01), and significant higher scores on affective empathy (*t*_(239)_ = −4.50, p < 0.001) and total empathy (*t*_(239)_ = −4.17, p < 0.001). No gender differences were present for reactive aggression, total aggression, and cognitive empathy. Reactive, proactive, and total aggression scores did not differ significantly between girls and boys within the aggression clusters. Only in the reactive-proactive aggression cluster, boys scored significantly higher than girls on proactive aggression (*t*_(60)_ = 3.19, p < 0.01). Independent samples t tests revealed that only in the low aggression cluster girls scored significantly higher than boys on affective empathy (*t*_(76)_ = − 2.80, p < 0.01) and total empathy (*t*_(76)_ = −2.88, *p* < .01). No significant differences were present between girls and boys within any aggression cluster on cognitive empathy.

**Table 6:** Aggression and empathy scores for boys and girls and results of gender comparisons within identified clusters and the total study sample

	Re pro AGG				Re AGG				Low AGG				Total sample			
	Girls (N = 16)		Boys (N = 46)		Girls (N = 39)		Boys (N = 62)		Girls (N = 32)		Boys (N = 46)		Girls (N = 87)		Boys (N = 154)	
	M	(SD)	M	(SD)	M	(SD)	M	(SD)	M	(SD)	M	(SD)	M	(SD)	M	(SD)
*Aggression (RPQ)*																
Reactive aggression	15.56	(3.29)	16.24	(4.24)	11.95	(2.37)	11.27	(2.72)	4.97	(1.88)	4.70	(1.76)	10.05	(4.75)	10.79	(5.42)
Proactive aggression	9.75^a^	(1.98)	12.15^b^	(3.85)	3.03	(2.10)	3.32	(2.25)	1.13	(1.12)	1.50	(1.49)	3.56^c^	(3.55)	5.42^d^	(5.20)
Total aggression	25.31	(4.18)	28.39	(6.54)	14.97	(3.48)	14.60	(2.10)	6.09	(2.52)	6.20	(2.51)	13.61^a^	(7.62)	16.21^b^	(9.69)
*Empathy (BES)*																
Cognitive empathy	34.75	(5.71)	33.98	(5.00)	37.43	(4.20)	37.21	(4.28)	38.50^a^	(3.73)	36.63^b^	(4.21)	37.33^a^	(4.50)	36.07^b^	(4.67)
Affective empathy	33.94	(5.87)	30.98	(5.55)	38.49^a^	(5.23)	34.95^b^	(8.22)	38.62^c^	(5.97)	34.09^d^	(7.71)	37.70^c^	(5.85)	33.51^d^	(7.51)
Total empathy	68.69	(8.78)	64.95	(7.29)	75.92	(11.04)	72.16	(11.04)	77.12^c^	(8.29)	70.71^d^	(10.52)	75.03^c^	(8.69)	69.8^d^	(10.31)

*RPQ* Reactive Proactive Aggression Questionnaire, *BES* Basic Empathy Scale, *re pro AGG* reactive-proactive aggression cluster, *re AGG* reactive aggression cluster, *low AGG* low aggression cluster

Subscripts (a, b) denote significant gender differences (p < 0.05)

Subscripts (c, d) denote significant gender differences (p < 0.01)

## Discussion

The present study extends previous research by evaluating the associations between cognitive and affective empathy and reactive and proactive aggression in adolescents. The study advances the field by investigating these associations in a sample of adolescents with elevated levels of aggression, and by comparing scores on cognitive and affective empathy between adolescents with distinct aggression profiles. Results showed that cognitive and affective empathy were significantly associated with proactive aggression, but not with reactive aggression. Cluster analysis yielded three clusters with meaningful profiles of reactive and proactive aggression that differed significantly on cognitive, affective, and total empathy scores. Within aggression clusters gender difference on empathy varied. Girls and boys within the reactive-proactive aggression cluster did not differ significantly on cognitive, affective, and total empathy. Whereas within the low and the reactive aggression cluster girls scored higher on affective empathy. Findings allow conclusions to be drawn on the interrelations of theoretically distinct aggression subtypes and different empathy facets.

With our first study question we investigated if cognitive and affective empathy are associated with reactive and proactive forms of aggression. In line with our hypothesis, we found negative associations between cognitive and affective empathy and proactive aggression. Our results confirmed other research indicating that proactive aggression [13] and bullying [45] are related to lower levels of empathy. Hence, according to our data lower scores on cognitive and affective empathy are associated with higher levels of aggression that is instrumental, organized, and motivated by the anticipation of reward. Our results further affirmed the hypothesis that reactive aggression is only marginally related to cognitive and affective empathy. We based our assumption on the specific characteristics of reactive aggression. Reactive-aggressive individuals are characterized by impaired emotion regulation [46] and reduced cognitive control under emotional stimulation [47]. Our results support a recent study showing that proactive but not reactive aggression is negatively associated with feelings of guilt in children [36]. Results of hierarchical regression analysis in the present study showed that cognitive and affective empathy explained additional variance of proactive aggression, beyond that afforded by reactive aggression, age, and gender. Of note, this was not the case for reactive aggression. This finding further confirmed our expectations regarding the associations between aggression subtypes and empathy facets. By showing that empathy is associated with proactive but not with reactive aggression, our study fosters a better understanding of the empathy-aggression relationship. Interestingly, regression analysis revealed a significant unique predictive value of affective empathy, while cognitive empathy did not uniquely add to the prediction of proactive aggression. This affirms the assumption of an empathy imbalance in proactive aggressive individuals [48]. Inconsistent findings on the association between empathy and aggression in previous studies are possibly due to insufficient differentiation of aggression subtypes and empathy subcomponents. Our findings indicate that the more specifically these concepts are defined and assessed the better their relationship can be understood. It is also possible that earlier studies reported only marginal associations between empathy and aggression, because samples with high levels of aggression were not included in a number of investigations, as has been criticized in recent reviews [11, 12]. To address this gap in the literature, we recruited a sample that was expected to show high levels of reactive and proactive aggression. In fact, reactive and proactive aggression scores of the reactive-proactive aggression and the reactive aggression clusters were comparable or even higher than in studies applying the same measure in antisocial juvenile populations [18, 44, 49]. Nonetheless, associations between proactive aggression and empathy in the present study were only of medium effect size. Lovett and Sheffield [12] argue that behavioral or experimental measures of empathy indicate stronger relationships with aggression. Thus, it would be interesting to assess if our results can be replicated using experimental paradigms that differentiate between cognitive and affective empathy. The development of such performance-based experimental paradigms is an important subject for future research.

The second aim of our study was to compare cognitive and affective empathy in adolescents with distinct aggression profiles. In summary, the reactive-proactive aggression cluster scored lower on affective, cognitive, and total empathy than the reactive aggression, and the low aggression cluster. The reactive aggression and the low aggression cluster did not differ on cognitive, affective, or total empathy. Our results are in line with previous studies that have reported empathy deficits in aggressive children and adolescents with disruptive behavior disorders using questionnaires, story vignettes, and experimental paradigms [50–53]. By using a statistical approach that allowed us to identify clusters of adolescents with meaningful aggression profiles, instead of comparing dichotomous study groups, our results support the utility and importance of the distinction between reactive and proactive aggression for research investigating the interrelation of empathy and aggression. Further, our study results confirm clinical assumptions about the relevance of empathy deficits in adolescents with high levels of reactive and proactive aggression. Surprisingly, although the low aggression and the reactive aggression clusters had almost identical mean scores on affective empathy, post hoc comparisons indicated that only the reactive aggression cluster differed significantly from the reactive-proactive aggression cluster, while the difference between the low aggression and the reactive-proactive aggression sclusters was only marginally significant. Of note, inspection of age and gender corrected estimated mean scores indicated that covariates had a larger influence on the group comparison between the low aggression and the reactive-proactive aggression cluster.

With our third study question we aimed to test whether girls and boys with similar aggression profiles differed on affective, cognitive, and total empathy. Our results showed that only in the low aggression cluster girls scored significantly higher on affective and total empathy. Although in each aggression cluster girls had higher scores than boys on affective and total empathy, descriptive statistics also indicated that differences on empathy measures between girls and boys within the reactive-proactive aggression cluster were smaller. This indicates that previously reported gender differences in empathy are less prominent in adolescents with aggressive behavior, especially when girls also show proactive aggression. Possibly, girls prone to show proactive aggressive behavior are less likely to experience positive reinforcement for empathic behavior and therefore show less affective empathy than non-aggressive girls. Further, the gender intensification theory by Hill and Lynch [54] indicates that girls and boys intend to act in ways that are consistent with gender-specific role expectations. Therefore, girls are usually encouraged to act emotionally responsive and show affective empathy, whereas boys tend to downplay such behaviors. Since institutionalized girls might have different gender role expectations, due to different peer group influences and a deviant socialization background, the direction of the hypotheses made by the gender identification might not apply for girls living in juvenile justice institutions. In line with our hypothesis and previous investigations in adolescents applying the same instrument, we did not find differences between girls and boys on cognitive empathy. Even for the entire study sample, gender differences on cognitive empathy were small. This finding further implicates the importance of the differentiation of cognitive and affective empathy subcomponents for future investigations.

### Limitations

The study has several limitations that should be mentioned. First, we used self-report to assess aggression and empathy. Using self-report for the assessment of the constructs under investigation has advantages and drawbacks. Social desirability is often a problem when self-report is used to measure aggressive behavior. Nonetheless, we used the RPQ in its self-report form for two reasons: (1) the self-report version has excellent psychometric properties [18, 44, 49] and (2) we expected adolescents to have the best knowledge of their general aggressiveness during the past six months. We chose the BES for similar reasons. The questionnaire has very good psychometric properties [10] and has been applied in multiple cultural settings and different languages [39, 40, 55]. Further, current research shows that self-report questionnaires capture empathy validly [56]. This appears comprehensible, since empathy is primarily an internal emotional process, rather than an observable behavior. Second, the differentiation of empathy into cognitive and affective components represent only one approach to disentangle the multilevel construct. Vachon and Lynam [26] recently introduced a new measure of empathy that assesses cognitive empathy and two subtypes of affective empathy (i.e., affective resonance and affective dissonance). The authors showed that aggressive behavior in healthy adults is more strongly associated with affective dissonance than with affective resonance. Hence, empathy questionnaires for children and adolescents might also need additional revision in this direction. Third, adolescents living in child welfare and juvenile justice institutions are characterized by a unique sociodemographic background [42]. Therefore, the results need to be carefully interpreted and replications of our findings are required before these can be generalized to other populations. Fourth, it is plausible that the age- and the gender-composition of the sample influenced the present findings. Both variables need to be considered as confounds in empathy research because differences have been reported [57]. We therefore controlled for the influence of gender and age in the hierarchical regression models and in the group comparisons. Of notice, hierarchical regression analysis showed that cognitive and affective empathy improved the prediction of proactive aggression beyond that afforded by age and gender in the regression models and gender distribution did not differ across derived aggression clusters. Nonetheless, statistical control is never an optimal replacement for experimental control. Thus, our results need to be verified in larger samples that allow gender and age specific investigations of our study-questions. Fifth, the present study is cross-sectional, which does not allow inferences about causality and the temporal stability of the associations indicated by our data. Sixth, sample sizes were different for gender comparisons within aggression clusters which reduced power for some of the group comparison.

## Conclusions

In conclusion, our study showed that the interrelation between cognitive and affective empathy and aggression subtypes depends partly on the specificity of the conceptualization of these constructs. Our results indicated that differences in cognitive and affective empathy are detectable between clusters of adolescents with meaningful aggression profiles of reactive and proactive aggression. It is tempting to speculate that heterogeneous findings in previous research are due to insufficient specification of the constructs under investigation and the characteristics of the study group. Given the limitations of the present study, future investigations are needed before conclusions for practical implications can be drawn. However, the present study may guide hypotheses for the development of specific treatment programs for aggressive adolescents. Results indicated that empathy is not equally associated with aggression subtypes. This is an important observation because it is often assumed that fostering empathy during clinical interventions reduces future aggressive behavior. According to our data, this assumption might be misleading, at least for primarily reactive aggressive individuals. Our results are in line with other studies emphasizing the importance of specific therapeutic approaches for different variants of aggressive adolescents [58, 59]. Further, our study showed that gender differences were less distinct in adolescents within the aggression cluster with high levels of reactive and proactive aggression. This finding might also help to advance clinical approaches for aggressive girls. Future longitudinal studies are necessary to understand the causality and the temporal stability of the relationship indicated by the present results.
